# Effect of the aggregation state of amyloid-beta (25-35) on the brain oxidative stress in vivo

**DOI:** 10.1371/journal.pone.0310258

**Published:** 2024-10-29

**Authors:** Anna Kozina, Goethe Herbert-Alonso, Alfonso Díaz, Gonzalo Flores, Jorge Guevara

**Affiliations:** 1 Instituto de Química, Universidad Nacional Autónoma de México, Mexico City, Mexico; 2 Facultad de Ciencias Químicas, Benemérita Universidad Autónoma de Puebla, Puebla, Mexico; 3 Instituto de Fisiología, Benemérita Universidad Autónoma de Puebla, Puebla, Mexico; 4 Facultad de Medicina, Universidad Nacional Autónoma de México, Mexico City, Mexico; Marche Polytechnic University, ITALY

## Abstract

Aggregation pathway of amyloid-*β* (25-35) in water affects the oxidative stress in the brain observed after administration of aggregated peptide in animals *in vivo*. Our studies on peptide aggregation *ex situ* prior to injection suggest that from the onset of peptide incubation in aqueous media, all samples exhibit the formation of fibril-like aggregates, characterized by a significant amount of *β*-sheets. This induces significant oxidative stress *in vivo* as observed for up to 60 min of peptide aggregation time. As the aggregation advances, the fibril-like aggregates become longer and intertwined, while the amount of *β*-sheets does not change significantly. An injection of such large, thick, and entangled aggregates in the animal brain results in a drastic increase in oxidative stress. This may be related to the number of activated microglia that initiate a sequence of inflammatory responses in the presence of large, highly interconnected fibrils.

## 1 Introduction

Amyloid-*β* peptides are the products of proteolytic cleavage of amyloid-*β* precursor protein (APP), a trans-membrane protein concentrated primarily in neuronal tissue [1]. The APP fragments amyloid-*β* (1-40) and amyloid-*β* (1-42) are the major components of the senile plaques of the Alzheimer’s Disease (AD) brain [2]. Both fragments are able to aggregate into fibril-like structures that become neurotoxic. Such amyloid accumulation is considered to be an early onset of AD [3].

Amyloid-*β* (25-35) is the shortest fragment of the amyloid-*β* (1-40/42) peptide that has a synthetic origin. It consists of 11 amino acids sequenced as *NH*_2_ − *Gly* − *Ser* − *Asn* − *Lys* − *Gly* − *Ala* − *Ile* − *Ile* − *Gly* − *Leu* − *Met* − *COOH*. Although it is not produced as a result of APP cleavage, it was found in the senile plaques of the AD brain as a residual fragment [4]. Nevertheless, amyloid-*β* (25-35) received much attention for being a short hydrophobic fragment of the full peptide that retains the biological properties of its full-length counterparts [5]. Similar to the full-length peptides, amyloid-*β* (25-35) produces a considerable inflammation response in the brain on intracerebral injection, and therefore it represents a toxic fragment of the full-length peptides [6]. Such toxicity is used in animal models of AD to induce memory impairment, cognitive decline, neuronal cell death, and increased oxidative stress, among other degenerative effects [6–10]. The toxicity of both full-length and truncated peptides is associated with their high ability to form insoluble aggregates, which has stimulated a large number of studies to elucidate the aggregation process. Gaining insight into the relationship between peptide aggregation and toxicity would facilitate the exploration of new paths toward aggregation inhibition *in situ* as a part of AD therapy [11, 12].

The aggregation pathway is governed in the first place by the formation of a peptide secondary structure, which *in vitro* is extremely sensitive to environmental conditions such as pH, temperature, sample pretreatment, type of solvent, medium ionic strength, etc. Conformations of a single molecule of amyloid-*β* (25-35) in the liquid environment are the random coil (disordered) or helix [13, 14]. The preferable conformation of oligomers depends mainly on the solvent polarity. For dimers, a diverse ensemble of well-organized molecules with high *β*-sheet content coexisting with disordered complexes has been reported [15]. For oligomers, computer simulations showed diverse results. For example, a direct assembly into highly ordered oligomers with a *β*-sheet core and hydrophobically interacting shell has been found [16–18]. On the other hand, it was shown that apolar solvents stabilized primarily a helical conformation, while polar solvents like water stabilized mainly random coils with a fraction of *β*-turns [13, 14]. To dissolve preexisting aggregates as well as stabilize disordered or helical conformations, a pretreatment of amyloid-*β* (25-35) by dissolution in fluorinated solvents is commonly done before aggregation experiments. Hexafluoroisopropanol (HFIP) and trifluoroacetic acid (TFA) at various concentrations in water are usually used. Pretreated amyloid-*β* (25-35) in neutral buffers (pH = 7.2-7.4) and polar solvents such as ethanol and dimethyl sulfoxide adopts mainly *β*-structures (*β*-sheets and *β*-turns) [17, 19–25], while at lower pH’s or concentrations, both the random coil and *β*-sheets were detected [19, 22, 25]. Buffer salinity and molarity increase the content of *β*-sheets and accelerate the formation of fibrils [21, 23, 25], while an addition of trifluoroethanol results in a mainly helical structure [26]. In water, mainly a disordered conformation was detected [17, 23, 25, 27], although the early reports also indicated the presence of mostly *β*-sheets [28, 29]. In the presence of a lipid membrane, the peptide showed preferably *β*-sheets [24, 30], which were related to the formation of amyloid pores made of 6-8 amyloid chains arranged mainly into a *β*-sheet structure. Recent reports [31] have confirmed membrane alteration in the presence of amyloid-*β* (25-35), emphasizing the significance of oligomers in peptide toxicity. Given these divergent reports, a reexamination of the amyloid-*β* (25–35) aggregation route in water with respect to aggregate secondary structure and morphology is required.

The correlation between amyloid-*β* (25-35) peptide aggregation *in vitro* and its toxicity in cell cultures was first reported by Pike *et al* [28]. The cell loss in the neuronal culture was investigated after the addition of pretreated amyloid-*β* (25-35) either freshly prepared or 7 days after its incubation in water. No significant difference was detected for each aggregation time, with about 64% cell loss found for each aggregation period, which was explained by the formation of *β*-sheets and visible insoluble aggregates. It was hypothesized that these two conditions were necessary to evoke cell toxicity since non-aggregated or non-*β*-sheets forming peptides were not toxic [29]. Specific interactions with the cell membrane were mentioned as a proposed mechanism of toxicity. The findings from the toxicity assessment conducted on rat pheochromocytoma cells (PC12) support the notion of oligomer toxicity. Specifically, the results indicated that the peptide had more toxicity when present in lower amounts of aggregates as opposed to a highly aggregated state [20]. However, there was no clear correlation between the toxicity and the amount of *β*-sheets on inhibition of *β*-sheet formation by mixing amyloid-*β* (25-35) with N-methylated peptides. The peptide incubated in phosphate buffered saline (PBS) was found to be more toxic for PC12 and SH-SY5Y cells than that incubated in phosphate buffer (PB) or water [21, 23]. This was explained by the presence of an increased *β*-sheet content after PBS incubation. Nevertheless, a different study found that the neurotoxicity in PC12 cells was more closely associated with the ability to aggregate than with a particular kind of secondary structure [26]. This finding may be connected to the increased toxicity shown after 48 hours of incubation as opposed to 24 hours [23]. Although the cell models allowed for the elucidation of important connections between the aggregation pathway and the toxicity, it is not always straightforward to establish a direct correlation between the cell toxicity *in vitro* and the more complex processes occurring in the brain. Hence, an analogous connection is required for experiments conducted *in vivo*.

In animal models, it has been observed that the direct injection of aggregated amyloid-*β* (25-35) into the brain leads to neurodegeneration, brain inflammation and neuronal death by apoptosis or necrosis [32–37]. One of the possible reasons for the neurodegeneration is attributed to the increased levels of oxidative stress observed in both adult and neonatal rats [6, 8–10, 38]. The administration of antioxidants either before or after the injection of amyloid-*β* (25-35) has been shown to significantly reduce this oxidative stress [39–45]. Activation of nitric oxide synthase (NOS) in the presence of amyloid-*β* (25-35) was shown to be responsible for the generation of nitric oxide (NO) reactive species, causing accelerated neuronal death [46, 47]. Inhibitors of NOS helped to reduce the inflammatory effects by suppressing the production of NO and inflammatory cytokines [48, 49]. Therefore, it is generally accepted that an inflammatory response due to the production of free radicals is one of the mechanisms of neuronal damage following amyloid-*β* (25-35) injection [50–52].

The majority of the *in vivo* studies report the effects caused by *in vitro* pre-aggregated amyloid-*β* (25-35), since it aggravates the neurodegenerative effects as compared to soluble amyloid fragments [53]. However, the systematic study of the correlation between the aggregation state of amyloid-*β* (25-35), its secondary structure, and the inflammatory neuronal response after its administration *in vivo* is lacking. Therefore, the present work aims to elucidate the impact of amyloid-*β* (25-35) aggregation state, namely the morphology and structure of the resulting aggregates, on the brain inflammatory response in terms of oxidative stress in an *in vivo* animal model. We demonstrate that during the initial stages of aggregation, there is evidence of the formation of small fibril-like aggregates with a mainly *β*-structure. This results in the first noticeable increase in oxidative stress in the animal brain. With time, the aggregates grow and interconnect, hence preserving the quantity of *β*-sheets as their secondary structure. Such aggregate growth and intertwining increase oxidative stress further, as characterized by lipid and protein peroxidation. We assume that the main cause of the second increase in oxidative damage is the degree of aggregation that triggers the activation of microglia, regardless of the amyloid secondary structure.

## 2 Materials and methods

### 2.1 Oxidative stress in vivo

#### 2.1.1 Animals

Adult male Wistar rats (280-320 g, n = 28) used in the study were provided by the animal facilities of the Faculty of Medicine of the National Autonomous University of Mexico (UNAM). The rats were housed in individual boxes at standard temperature and humidity conditions with 12-hour light-dark cycles. Food and water were readily available. The experiments were conducted in accordance with “The Guidelines for the Use of Animals in Neuroscience Research” from the Society for Neuroscience and “Guide for the Care and Use of Laboratory Animals of the Mexican Council for Animal Care” NOM-062-ZOO-1999. We made all the necessary efforts to minimize the number of animals. It was ensured to cause the minimum amount of pain and/or discomfort to the animals.

#### 2.1.2 Amyloid-*β* (25-35) injection

The animals were divided into six groups (4 animals per group) according to the aggregation time of the injected amyloid-*β* (25-35) and a control group. Amyloid-*β* (25-35) (purity ≥97%, liophilized from 0.1% TFA, Merck, USA) was dissolved in sterile water at a concentration of 1 mg/mL and left at room temperature to aggregate for 0, 10, 30, 60 min, 24 and 48 hours. After the indicated time, 1 *μ*L of the sample was injected into anesthetized animals (sodium pentobarbital, 75 mg/kg, IP) placed onto a stereotaxic frame (Stoelting Co., Wood Dale, USA). The stereotaxic coordinates for a bilateral injection in the hippocampus were: -4.2 from the bregma, -3.1 from the midline, and -3.0 below the dura, following Paxinos&Watson [54]. The control group was injected with the same volume of sterile normal saline. After the surgery, the animals were returned to their cages, where they were monitored constantly during the recovery.

#### 2.1.3 Lipid peroxidation assay

Four hours after injection, the animals were anesthetized with sodium pentobarbital (40 mg/kg) to minimize animal distress and then sacrificed by decapitation. The brains were extracted, washed with cold normal saline, and the hippocampi were dissected. Then, these were homogenized in 2 mL of the ice-cold PBS (0.1 M, pH = 7.4). The homogenates were centrifuged at 12500 rpm at 4°C. Then, 1 mL of each supernatant from the hippocampal tissue was added to 4 mL of a mixture of chloroform and methanol (2:1, v/v). The samples were placed on ice and stirred for 30 min in the dark. The upper phases (methanol) were discarded, while the lower phases (chloroform) were used to determine the formation of lipid-soluble fluorescent compounds as described previously [42, 55, 56]. The fluorescence of chloroform was determined with a PerkinElmer LS50-B luminescence spectrophotometer (PerkinElmer, USA) at the excitation and emission wavelengths of 370 and 430 nm, respectively. The sensitivity of the equipment was adjusted to a fluorescent signal of 140 fluorescence units (FU) with a standard solution of quinine (0.001 mg/mL of quinine in 0.05 M sulfuric acid). The results were expressed in relative fluorescence units (URF) per mg of protein [56].

#### 2.1.4 Nitric oxide assay

The supernatants of hippocampal tissues were used to quantify the concentration of nitrite (${\text{NO}}_{2}^{-}$), a stable breakdown product of NO. The Griess technique [9] was used to measure ${\text{NO}}_{2}^{-}$ (Griess Reagent System, Promega, USA). Absorbance was measured with a 540 nm filter with a Beckman DU-64 UV/Vis spectrophotometer (Beckman Coulter, USA). Results were expressed as micromoles of nitrite per milligram of protein (*μ*M of ${\text{NO}}_{2}^{-}/\text{mg}$ of protein).

### 2.2 Amyloid-*β* (25-35) aggregation

Amyloid-*β* (25-35) (purity ≥97%, liophilized from 0.1% TFA) and Thioflavin-T (ThT) were purchased from Merck (USA) and used as received. In all the experiments, deionized Milli-Q water (resistivity of 18.2 MΩ ⋅ cm) was used. For sample preparation, 1 mg of amyloid-*β* (25-35) was dissolved in 1 mL of water, and then aliquots of 50 *μ*L (each containing 50 *μ*g of the peptide) were distributed in Eppendorf tubes and stored at -20^∘^ C until further usage.

#### 2.2.1 ThT fluorescence spectroscopy

First, 1900 *μ*L of 50 *μ*M ThT aqueous solution were pored in a quartz 10-mm-path fluorescence cell (Hellma, Germany). The sample was measured with a Cary Eclipse fluorescence spectrometer (Agilent, USA) equipped with a rapid mix accessory and a thermostatted cell holder. Excitation and emission wavelengths were set at 440 and 480 nm, respectively. Then, 100 *μ*L of a 1 mg/mL peptide were rapidly added to the cell containing the ThT solution, resulting in a molar peptide concentration of 47 *μ*M (50 *μ*g/mL). The sample was manually mixed and then placed immediately into the spectrometer for measurement with maximum magnetic stirring. Emission was recorded at 20°C every 30 s for the first 10 min and after that at longer time intervals up to a few hours. To check the effect of sedimentation, in one of the experiments, the stirring was turned off during the first 45 min and then turned on again to complete the experiment.

#### 2.2.2 Optical microscopy

Optical microscopy images were taken with an AxioImager optical microscope (Zeiss, Germany) with a GFT shift-free filter set. Aggregated samples were observed in a 1-mm-path quartz cuvette (Hellma, Germany) at different aggregation times. Aggregation conditions were maintained equal to those for ThT fluorescence spectroscopy; therefore, fluorescence of ThT was detected.

#### 2.2.3 Circular dichroism (CD) spectroscopy

CD spectra were recorded with a Jasco J-1500 CD spectrometer (Jasco, Japan). 300 *μ*L of aqueous peptide solution at a concentration of 0.17 mg/mL was placed in a 1-mm-path quartz cuvette (Hellma, Germany) and left to incubate at 20°C for 10 min, and 1, 2, 3, and 24 hours. At certain incubation times, a triplicate of spectra was recorded in the wavelength region of 190−250 nm at a scan rate of 50 nm/min. The high voltage was varied automatically below 400 V. The spectra were corrected by the medium baseline.

#### 2.2.4 Fourier transform infrared spectroscopy (ATR-FTIR)

The ATR-FTIR spectra were collected using a Nicolet iS50 FTIR spectrometer (Thermo Fisher Scientific, USA) equipped with a calcium fluoride crystal accessory. 50 *μ*L of aqueous solution of amyloid-*β* (25-35) (1 mg/mL) were incubated in an Eppendorf tube at 20°C for 48 hours. At certain incubation time intervals, an aliquot of 3 *μ*L was taken from the tube and placed on the crystal for measurement. Spectra were recorded in the region of 4000-400 cm^−1^ with a resolution of 4 cm^−1^ over 64 scans and corrected for the background. Each measurement was repeated three times.

#### 2.2.5 Electron microscopy

For transmision electron microscopy (TEM), 50 *μ*L (1 mg/mL) of peptide were added to 500 *μ*L of either water or PBS and separated into five equal parts. The first sample was prepared immediately, while the rest four samples were left at room temperature to aggregate for 10 min, and 1, 24 and 48 hours. Then, 5 *μ*L of each sample were dropped on a 200-mesh copper grid previously coated with 2% Collodion solution (EMS, USA). The grid was blotted and dried at room temperature in a vacuum. Then, the sample was stained with 2% uranyl acetate solution and washed with water. Samples were visualized with a JEOL JEM-2010 FEG microscope (Jeol, Japan) at 200 kV. Scanning transmission electron microscopy (STEM) images were obtained with a JSM-7800F electron microscope (Jeol, Japan) at an accelerating voltage of 15 kV.

## 3 Results and discussion

**Fig 1:**
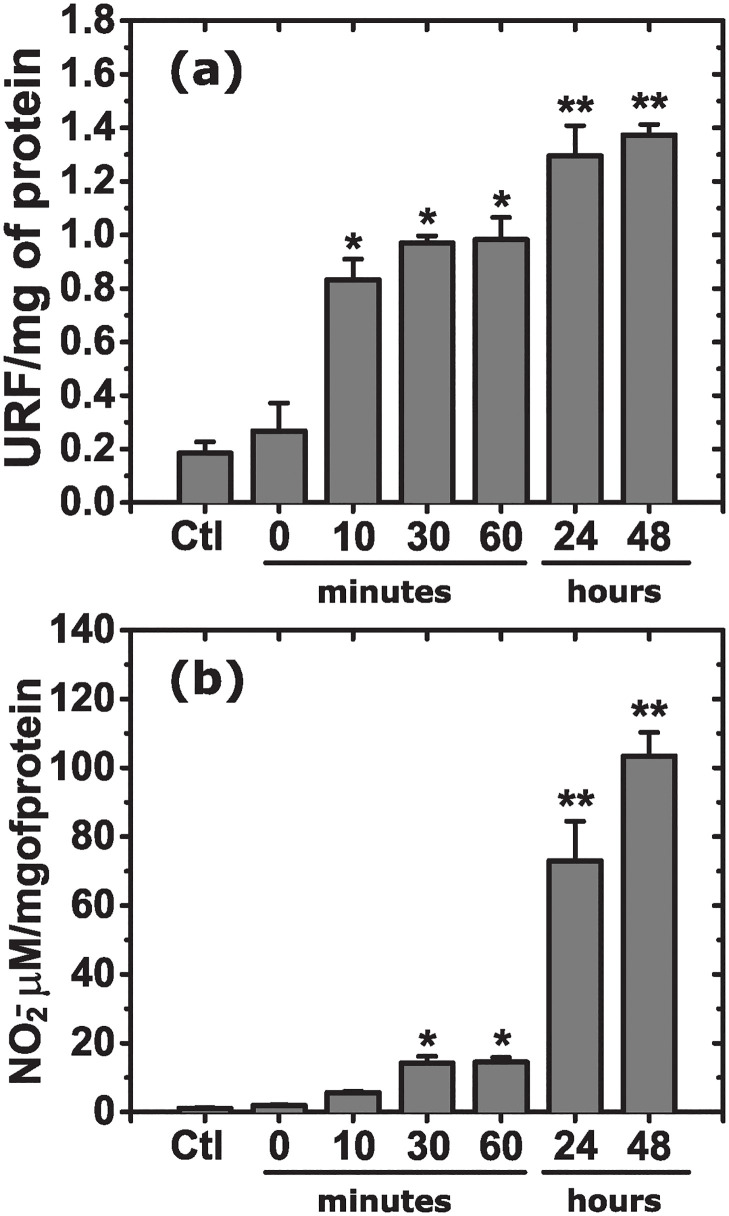
Levels of lipid peroxidation (a) and nitrite (b) for the animal groups according to the aggregation times of amyloid-*β* (25-35) before injection. *P < 0.05, **P < 0.01 as compared to the control group (one-way ANOVA and Bonferroni post-hoc test).

Fig 1 shows levels of lipid peroxidation and nitrite for the studied groups corresponding to different aggregation times of amyloid-*β* (25-35) before injection. With increasing amyloid-*β* (25-35) aggregation time, the levels of both lipid peroxidation and nitrite increase, indicating an overall increase in oxidative stress. Nevertheless, the difference in the levels of the oxidative species during the first hour of incubation (10, 30, and 60 min) is not significant between them, while it is significant as compared to the control and 0 hours-incubation groups. The most significant difference appears between the short- and long-term peptide aggregation groups, e.g., 60 min and 24 hours, while the difference between 24 and 48 hours is less pronounced. Such a correlation between the time of peptide aggregation and the brain response *in vivo* suggests that the structural changes in the amyloid-*β* (25-35) on aggregation may be an origin of the peptide toxicity. The first possible reason is the change in the peptide secondary structure. Therefore, kinetic studies of peptide aggregation were performed to elucidate the evolution of the secondary structure during peptide incubation.

**Fig 2:**
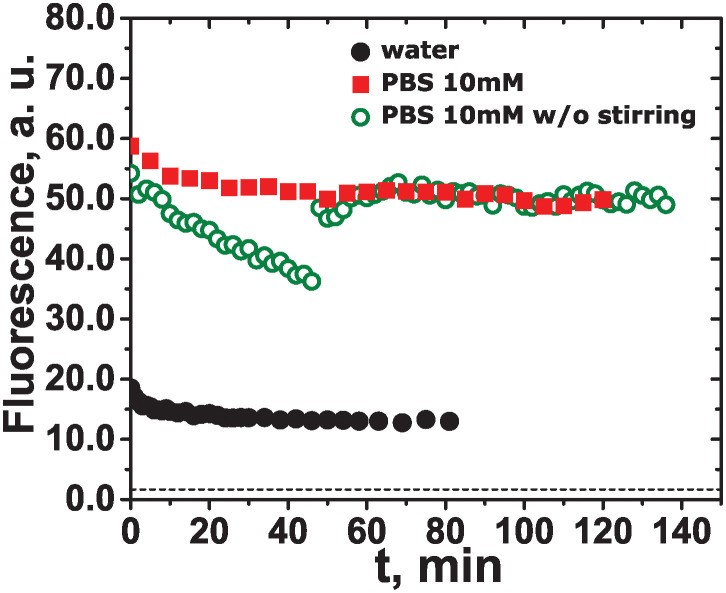
Fluorescence intensity of ThT solution as a function of time for amyloid-*β* (25-35) aggregating in water (filled circles), in PBS (10 mM) (squares), and in PBS (10 mM) without stirring during the first 45 min (open circles). The black dashed line indicates ThT fluorescence in the absence of peptide.

Fig 2 shows the kinetics of amyloid-*β* (25-35) aggregation in water and in PBS (10 mM) as measured by ThT fluorescence. The ThT fluorescence intensity is very low in the absence of the peptide and increases significantly in its presence. ThT is a marker that binds specifically to amyloid fibrils in the *β*-sheet conformation, which results in a shift in its emission maximum [57]. This occurs because ThT molecules align with their long axes parallel to the long axis of the fibril. Thus, ThT fluorescence enhancement upon binding to amyloid is possible only in the presence of *β*-sheets. Such an enhancement is observed for both water and PBS, suggesting the formation of *β*-sheets immediately after peptide addition. A more pronounced increase in ThT fluorescence for PBS may reflect either a larger overall content of *β*-sheets or a larger contribution of a certain type of *β*-structures, to whom ThT might be more affine [58]. For both media, the fluorescence is almost constant in time, with a small decrease at initial times. The fluorescence plateau suggests that the aggregation is extremely fast, so that the initial sigmoid-like behavior is not observed. Therefore, the *β*-sheet secondary structure is formed rapidly, and its content does not change over the experimental time interval. Previous works on the aggregation kinetics of HFIP or TFA pretreated amyloid-*β* (25-35) in water, PBS, and acetonitril indicated aggregation tendencies affected by the solvent used [17, 23, 27]. Thus, amyloid-*β* (25-35) incubated in water and in a mixture of water and acetonitril resulted in mainly disordered conformations without *β*-sheets. This was also reflected in the constant low fluorescence during the ThT assay. In contrast, the incubation in PBS promoted the formation of *β*-sheets, and an increased fluorescence of ThT was observed. The aggregation pathway observed in Fig 2 is governed mainly by the formation of *β*-sheets rather than hydrophobic interactions, which is more pronounced in PBS as compared to water. We suggest that an increase in the medium ionic strength due to the presence of ions in PBS may be responsible for a promoted *β*-sheet formation or for a preferential type of it, which is possibly assisted by alteration in electrostatic and hydrogen-bond interactions.

**Fig 3:**
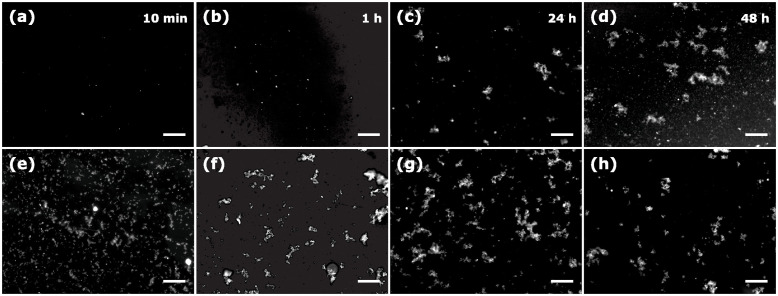
Fluorescent microscopy images of aggregated amyloid-*β* (25-35) at different times in water (upper row) and PBS (lower row): (a), (e) 10 min; (b), (f) 1 hour; (c), (g) 24 hours; and (d), (h) 48 hours. Scale bars = 100 *μ*m.

To assign the initial small fluorescence decay, an additional experiment in PBS without stirring was carried out (open circles in Fig 2). The sample was not stirred for up to 45 min after the peptide addition to the ThT solution, and then the stirring was switched on and the sample was further measured for up to 140 min. One may notice that during the initial 45 min there is a pronounced fluorescence decay that is almost linear. This occurs due to the sedimentation of the large aggregates. Once the stirring started, the aggregates were distributed in the entire sample volume, which increased the overall sample fluorescence that stayed constant while the stirring was maintained. This experiment emphasizes the importance of vigorous sample stirring when heterogeneous systems like aggregated amyloid peptides are studied. The formed aggregates were noticeable to the naked eye and could be visualized under fluorescent and electron microscopes. Fig 3 shows the aggregates formed in water and PBS as seen under a fluorescent microscope.

**Fig 4:**
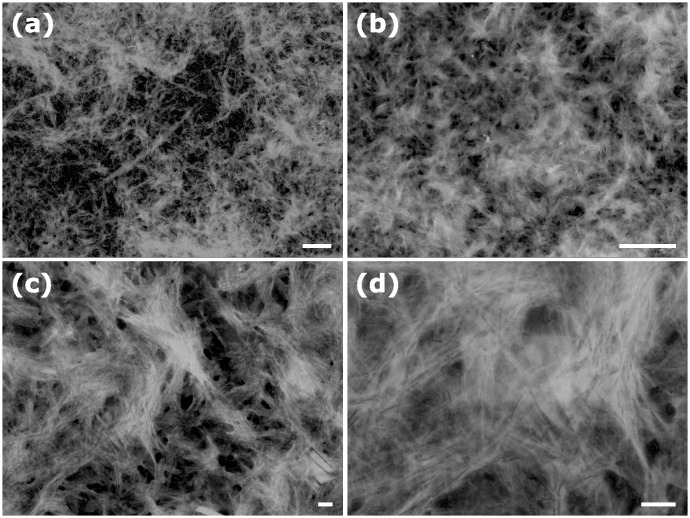
SEM micrographs of aggregated amyloid-*β* (25-35) in water for 24 hours. Scale bars: upper row = 1 *μ*m, lower row = 100 nm.

Aggregation occurs in both water and PBS. However, the process of aggregation in water is considerably slower compared to that in PBS. Aggregates in water are barely visible at aggregation times of 10 min and 1 hour, while their formation in PBS becomes evident already after 10 min. The aggregates increase in size and interconnect over time, as observed in the 24-hour images. After 48 hours in PBS, all the aggregates exhibit a substantial size and a fluffy texture. Conversely, in water, a significant quantity of small aggregates remains clearly observable. Therefore, it can be inferred that PBS accelerates the process of aggregation in comparison to water. On closer observation, large aggregates seem to be made of smaller ones; however, a fibrillar structure is not distinguishable. Fig 4 shows STEM images of the aggregates in water shown above. On observation by STEM, it is noticeable that the material is fibrillar, forming a large interconnected network. Larger magnifications allow for the distinction of long and thin individual fibers, confirming the formation of fibrillar structures.

**Fig 5:**
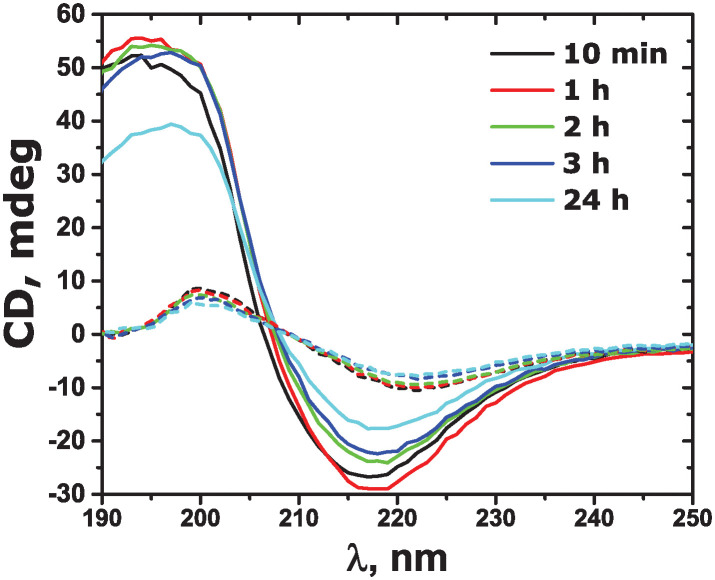
CD spectra taken at different times of amyloid-*β* (25-35) incubation in water (solid lines) and in PBS (10 mM) (dashed lines) as given in the legend.

To further validate the observations of the secondary amyloid structure as detected by ThT fluorescence, the aggregation kinetics were followed by CD and ATR-FTIR spectroscopies. CD spectra of amyloid-*β* (25-35) at different aggregation times are shown in Fig 5. In water, there is a negative minimum at 218 nm and a positive maximum at 197 nm for all the aggregation times, which suggests the presence of *β*-structures for all the aggregation times [22]. The spectra minima in PBS are shifted to 222 nm as compared to water, while the maxima are at 200 nm, which may be indicative of *β*-structures with a different type of twist [59]. Spectra analysis using BeStSel resulted in the secondary structures as given in Table 1.

**Table 1:** Relative content of secondary structures (%) from CD spectra.

	10 min	1 h	2 h	3 h	24 h
	water				
*α*-helix	6.5	6.5	6.5	0	0
*β*-sheets antiparallel	93.5	93.5	93.5	100.0	100.0
*β*-sheets parallel	0	0	0	0	0
disordered	0	0	0	0	0
	PBS				
*α*-helix	11.0	16.2	22.0	16.7	16.9
*β*-sheets antiparallel	48.0	48.0	39.8	39.0	33.7
*β*-sheets parallel	41.0	35.8	38.1	44.3	49.4
disordered	0	0	0	0	0

**Fig 6:**
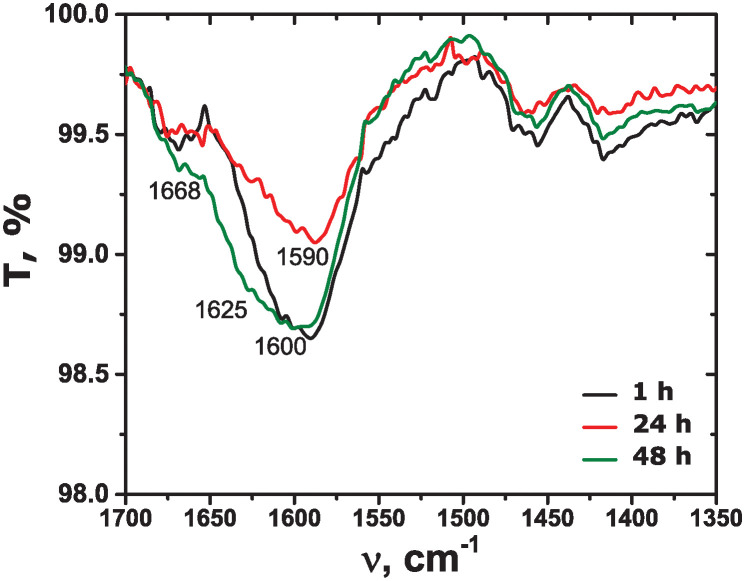
ATR-FTIR spectra of amyloid-*β* (25-35) at different times of aggregation in water, as given in the legend.

The *β*-sheet secondary structure is indeed predominant for all the incubation times. While the antiparallel *β*-sheets are mainly formed in water, in PBS, a significant contribution of parallel *β*-sheets is detected. In addition, a larger contribution of helical structure is evident in PBS. This conformational difference is reflected in the small shifts in CD spectra minima mentioned above. The *β*-sheet content is rather high from the very beginning of aggregation in both water and PBS. While it increases slightly with time in water, it stays practically constant in PBS. Both observations are in agreement with the results observed by ThT fluorescence kinetics. Interestingly, in PBS there is still a contribution of helical structure even after 24 hours of aggregation, while in water it converts completely to the *β*-sheets. Our results are different from the previous reports, where mainly disordered conformation in water was found [17, 23, 25, 27]. This might stem from the accuracy of the determination algorithms used by different types of software. ATR-FTIR spectra of the amyloid aggregated in water are shown in Fig 6. All the spectra show similar features. There is a broad band around 1590 cm^−1^ and a small one around 1668 cm^−1^. Both bands indicate the presence of the *β*-structure in the amide I band [62], although the small band may also correspond to turns and *α*-helices [30, 60]. Similar bands were previously observed for amyloid-*β* (25-35) aggregated in different environments [22, 23, 61]. A small shift to lower frequencies of the strong band may be related to the formation of fibrils and their different morphology as compared to native *β*-sheets [23, 62]. No significant spectra change is observed from 1 to 24 hours of aggregation, while at 48 hours the band at 1590 cm^−1^ slightly shifts to around 1600 cm^−1^ and broadens. An emerging band at 1625 cm^−1^appears, which may be related to the appearance of larger or thicker fibrils at such a long aggregation time. ATR-FTIR spectra are in agreement with the results obtained by ThT fluorescence and CD: the amyloid-*β* (25-35) secondary structure on aggregation in water is mainly the *β*-sheets, the fibril formation is extremely fast without further significant alteration of the secondary structure.

**Fig 7:**
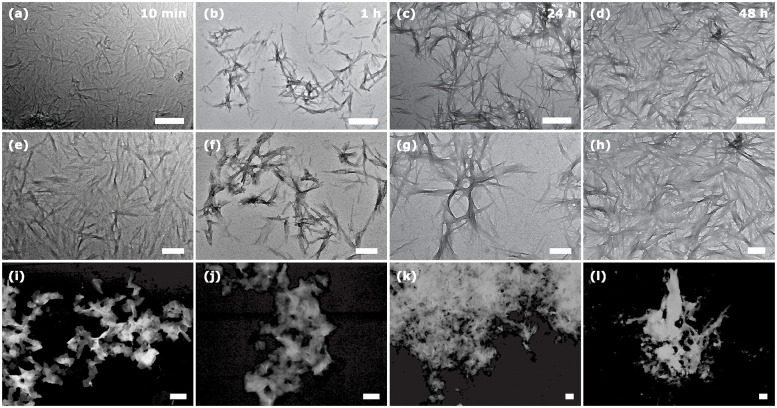
TEM/STEM micrographs of aggregated amyloid-*β* (25-35) at different times. In water: (a), (e) 10 min; (b), (f) 1 hour; (c), (g) 24 hours; and (d), (h) 48 hours. In PBS: (i) 10 min; (j) 1 hour; (k) 24 hours; and (l) 48 hours. Scale bars: upper row = 500 nm, medium and lower rows = 200 nm.

Since the secondary structure of the fibrils is mainly *β*-sheets, and the fibrils are formed practically immediately in water, they are responsible for the initial fast increase in the oxidative stress *in vivo* observed for as short as 10 min peptide incubation time in Fig 1. Then, the *β*-sheet content grows only slightly with time, which is in agreement with a moderated increase in the oxidative stress detected from 10 to 60 min. However, the further increase in oxidative stress observed from 60 min to 24 hours cannot be explained by the influence of the secondary *β*-sheet structure alone, since by 60 min in water, almost the entire peptide is in the *β*-sheet conformation. One potential explanation for the significant rise in oxidative stress could be attributed to changes in the size and shape of peptide aggregates. Therefore, we used electron microscopy to visualize the aggregates formed at different aggregation times, Fig 7. The micrographs of show that aggregates are already present at the very beginning of incubation (10 min) in water. The aggregates in water are fibril-like and are below 500 nm in length. In 1 hour of aggregation, intertwined fibrils appear, although the length of individual fibrils is still about 200−300 nm. On further aggregation at 24 hours, the fibrils become noticeably longer, with thicker interlaced regions. Individual fibrils are less noticeable, suggesting that thicker fibrils are formed by the aggregation of several individual fibrils along their large axes. This aggregation results in a dense network 48 hours after the incubation starts. The amyloid aggregates in PBS (Fig 7i–7l) also appear as early as 10 min of incubation time. However, the aggregate morphology is noticeably different. The fibrils are much shorter and organized in larger sheets or rod-like structures. These rods (sheets) are randomly interconnected, forming larger aggregates with a rather ramified morphology. In contrast to aggregates in water, the ones in PBS exhibit stronger attractitive inter-fibrillar interactions, preventing the detection of separate fibrils. As incubation time passes, such ramified aggregates become larger (such as in 24 hours) and more compact (such as in 48 hours). Therefore, while the medium only marginally affects the secondary structure, the morphology of inter-fibrillar aggregates differs significantly between water and PBS, underlining the significance of the peptide incubation medium.

The results from TEM agree with those obtained by ThT fluorescence, CD, and ATR-FTIR: the peptide aggregates from the beginning without a significant further change in its secondary structure; however, the size and morphology of the large aggregates do change. The most noticeable difference is detected between 1 and 24 hours of aggregation when thick and highly intertwined fibrils (ramified large aggregates) appear in water (PBS). Such a difference may be correlated with a drastic increase in the oxidative stress observed in Fig 1. We suggest the following possible explanation for this observation. It is known that insoluble fibrillar amyloid-*β* in the brain is internalized and phagocytosed by microglia [63, 64], which is the process of natural clearance of amyloid plaques. Whether the fibrils can later be degraded intracellularly remains open. The activated microglia initiate a sequence of inflammatory responses such as the production of proinflammatory cytokines, TNF_*α*_ and IL1-*β*, which stimulate the appearance of reactive oxygen species and nitric oxide, characteristic markers of neuronal death [65–67]. The number of microglia participating in the process of clearance is proportional to the size of the plaques, as is the inflammatory response [67]. For example, small amyloid aggregates injected into the rat brain can be completely cleared 28 days after the injection [68]. However, chronic inflammation appears when microglia cannot keep up with the aggregate clearance [69]. Therefore, we hypothesize that the presence of larger and more interconnected aggregates poses a significant challenge for microglia in terms of their removal, thereby leading to a stronger oxidative response, regardless of the peptide secondary structure.

## 4 Conclusions

Aggregation of amyloid-*β* (25-35) peptide in aqueous media plays a key role in the induction of an inflammatory response on its injection *in vivo*. Aggregation of amyloid-*β* (25-35) peptide in water begins with the initial formation of fibril-like aggregates with a significant amount of *β*-sheets as a secondary peptide structure. As the aggregation proceeds, the fibrils grow longer and thicker, interconnecting at the same time. In water, this process is accompanied by a further small increase in *β*-sheet content with a mainly antiparallel structure. In PBS, a significant amount of *α*-helix and parallel *β*-sheet are present. The peptide toxicity *in vivo* as seen by oxidative species increases with peptide aggregation time in two steps. First, it increases almost immediately up to 60 min of aggregation, which is related to the fast formation of individual fibrils. Then, even stronger brain inflammation is observed after administration of the more aggregated peptide, as indicated by the higher levels of NO species and lipid peroxidation. This second increase stems from the highly entangled fibrils and overall larger aggregates, which present difficulties for microglia in effectively clearing such structures. Our results may be useful for the elaboration of new strategies in AD therapies, considering brain inflammation response as a consequence of amyloid aggregation.
